# Enhancing Smallholder Access to Agricultural Machinery Services: Lessons from Bangladesh

**DOI:** 10.1080/00220388.2016.1257116

**Published:** 2016-11-28

**Authors:** Khondoker A. Mottaleb, Dil Bahadur Rahut, Akhter Ali, Bruno Gérard, Olaf Erenstein

**Affiliations:** * Socioeconomics Program, International Maize and Wheat Improvement Center (CIMMYT), Texcoco, Mexico; ** International Maize and Wheat Improvement Center (CIMMYT), Socioeconomics Program, National Agricultural Research Center, Islamabad, Pakistan; † Sustainable Intensification Program (SIP), International Maize and Wheat Improvement Center (CIMMYT), Texcoco, Mexico

## Abstract

Resource poor smallholders in developing countries often lack access to capital goods such as farm machinery. Enabling adequate access through machinery services can thereby significantly contribute to food security and farm incomes. At the core of the service provision model is the lead farmer, who makes the initial investment in agricultural machinery, and provides services to others on a fee-for-service basis. Profiling the lead farmers can thereby provide important lessons and scaling implications. The present paper provides a case study of Bangladesh, using primary data to characterise the lead farmers. General education, credit availability and risk taking attitude play significant roles in whether or not a farm household will be a lead farmer in Bangladesh.

## 1. Introduction

Agricultural machinery can provide important benefits and has seen various development initiatives in developing countries, yet the pace of agricultural mechanisation overall and access to agricultural machinery by smallholders in many developing countries, particularly in sub-Saharan Africa, remains quite low. For example, manual (for example hoe) agriculture still prevails in large swathes of Africa and few have access to oxen and/or tractors for agricultural field operations (*Food and Agriculture Organization [FAO]*, 2008; *Hatibu*, 2013). Wider access to agricultural machinery in these countries, where agriculture contributes nearly 50 per cent to the countries’ GDP (*FAO*, 2008), can not only reduce the drudgery of women and children, but it can also increase agricultural productivity, and thus improve the livelihoods of the millions of resource-poor farm households. The question arises as to how to ensure wider access to agricultural machinery by smallholders in resource-poor developing countries?

Extending an Indian or Chinese style agricultural mechanisation drive that depends on heavy government subsidy and investment in rural infrastructure appears challenging in most poverty-stricken agriculture-dependent developing countries, considering the heavy financial burden and potential government failure (*Baudron et al.*, 2015; *Sims, Kienzle, Cuevas, & Wall*, 2006). Instead, agricultural mechanisation drives that require less investment yet ensure wider access to farm machinery even by marginal farmers appear more promising (following the Bangladesh, Thailand, Sri Lanka and Vietnam model, for example, *Justice & Biggs*, 2013; *Mottaleb & Krupnik*, 2015). Contrary to the widely-observed owner-operator system in the developed countries, in Bangladesh and other South and East Asian countries, there are often service providers that make the bulky capital good accessible on a divisible rental basis. These are often lead farmers, who initially invest in scale-appropriate agricultural machinery, and then provide services to other farmers on a fee-for-service basis (*Justice & Biggs*, 2013; *Mottaleb, Krupnik, & Erenstein*, 2016). It thus ensures wider access to agricultural machinery even by marginal farmers, who cannot purchase the machines due to the high financial burden but are ready to purchase services paying the service charge to the lead farmers on a commercial basis. For example, in Bangladesh, while more than 80 per cent of the agricultural land is now cultivated mostly using two-wheeled tractor-driven Power Tillers (PTs), only one in 30 farm households owns a PT (*Justice & Biggs*, 2013). This means most of the farmers have access to PT services, where PT owners provide PT services. Similar to PT owners, there are a large numbers of irrigation pump owners and rice and wheat thresher machine owners who provide irrigation and threshing services to client farmers on a fee-for-service basis (for example, International Development Enterprises [*iDE]*, 2012; *Mottaleb & Krupnik*, 2015).

Bangladesh’s achievement of ensuring wider access to scale-appropriate agricultural machinery even by resource-poor smallholders has been well reported (*Baudron et al.*, 2015; *Kienzle, Ashburner, & Sims*, 2013). Existing studies, however, seldom focus on the lead farmers, who are the mainstay of the service provision process: initially investing in scale-appropriate machinery, taking all the financial risks, and providing services to client farmers on a fee-for-service basis. It has been suggested to extend the Bangladeshi-style agricultural mechanisation elsewhere (for example, *Baudron et al.*, 2015; *Kienzle et al.*, 2013), which makes it imperative to understand the characteristics of the lead farmers cum service providers. Particularly, it is necessary to understand who invests in agricultural machinery and provides services to others, and how the mutually agreed service charges are set. This paper explores these two questions for the case of Bangladesh using primary data.

The rest of the paper is organised as follows: Section 2 briefly describes the process of overall agricultural mechanisation in Bangladesh since the 1970s, highlighting the role of market liberalisation under which a number of tariff and non-tariff barriers were removed, facilitating the import of mainly Chinese-made diesel engines and machinery; Section 3 describes the sampling process, the data used, and specifies the econometric models; Section 4 presents the major findings and Section 5 concludes.

## 2. Agricultural mechanisation in Bangladesh: role of market liberalisation

Agricultural mechanisation in Bangladesh was initiated under strong government initiatives, similar to many other developing countries in Asia and Africa (for example, *Mrema, Baker, & Kahan*, 2008). Under the ‘Mechanised Cultivation and Power Pump Irrigation’ scheme of 1950–1951, the government introduced four-wheeled tractors for field preparation and smaller engines for irrigation (*Ahmmed*, 2014). During 1960–1965, the government distributed 2238 pumps, 200 tractors, and 13,828 sprayers to farmer groups, and established two workshops at the government level to produce and repair agricultural machinery (*Ahmmed*, 2014). After independence in 1971, the Government of Bangladesh (GOB) continued strong support to facilitate agricultural mechanisation. The GOB established large-scale irrigation facilities by establishing Deep Tube Wells (DTWs) and renting out Low Lift Pumps (LLPs) to farmer groups. In addition, through the Bangladesh Agricultural Development Corporation (BADC), the GOB provided diesel for irrigation pumps at a subsidised rate of 75 per cent (*Hossain*, 2009). By the end of 1978, the BADC had rented out and managed a total of 9000 DTWs and 35,000 LLPs (*iDE*, 2012).

With the change in the political regime, and partly because of the heavy financial and management burdens of managing the large number of irrigation pumps, eight years after independence, the GOB embraced a market liberalisation policy and gradually started selling pumps and DTWs to farmer cooperatives and to individual farmers (for example, *Mottaleb & Krupnik*, 2015). Initially, privatisation progressed slowly, but it soon gained momentum when the government removed a number of tariff and non-tariff barriers on the imports of Chinese-made, comparatively-cheap, small diesel engines and other agricultural equipment, such as two-wheeled tractor driven power tillers (PTs). The liberalisation was part of an emergency response to the aftermath of a devastating cyclone in 1988, which took a major toll on human lives as well as draught animals used for land preparation – the resulting draught shortage estimated to be equivalent to some 132,000 tractors (Government of Bangladesh [GOB], 1989).

Anticipating food-shortages in the cyclone’s aftermath the government took two major steps. First, in 1988 it removed import tariffs on small diesel engines and reduced import tariffs on small tractors used for irrigation and land preparation; and second, in 1989 it abolished non-tariff barriers by eliminating the Standard Committee. At the time, the Standard Committee of Bangladesh was responsible to ensure the quality of imported machinery, including agricultural equipment. The committee maintained an exclusive list of specific types and brands of irrigation and land preparation machinery which were allowed to be imported – and non-listed machinery types were not allowed to be imported (*Gisselquist, Nash, & Pray*, 2002). The committee only allowed the import of high quality but costly Japanese machinery (*Justice & Biggs*, 2013). Chinese-made machinery, which was comparatively more affordable, was treated by the committee as low quality and not allowed. The abolishment of the Standard Committee facilitated the import of Chinese-made cheap low horsepower (HP) diesel engines and PTs (*Gisselquist et al.*, 2002; *Justice & Biggs*, 2013; *Mottaleb et al.*, 2016). By 1995, the GOB completely abolished tariffs on PT imports (*Hossain*, 2009).

**Figure 1.**
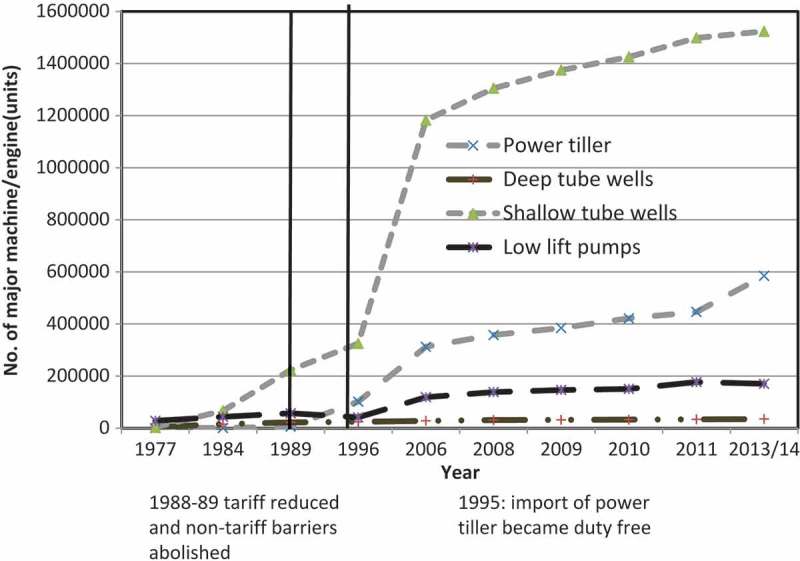
Agricultural machinery stock in Bangladesh, 1977–2013. *Sources*: iDE(2012); Ahmmed (2014)

Market liberalisation through the removal of tariff and non-tariff barriers resulted in a dramatic increase in imports of one-cylinder diesel engines for irrigation from India and China, and PTs from China (Figure 1). For example, the number of shallow tube wells (STWs) increased by 200 per cent from 93,000 in 1982 to 260,000 in 1990 (*iDE*, 2012). A total of 1.63 million irrigation units (STWs, DTWs and LLPs – *Bangladesh Agricultural Development Corporation [BADC]*, 2013) are now engaged for irrigating nearly 55 per cent of the cropland (*BBS*, 2011a). At present, an estimated half a million PTs, most of Chinese origin (*Ahmmed*, 2014; *iDE*, 2012), are engaged in preparing 80 per cent of Bangladesh’s eight million hectares of net cropland^1^ (BBS, 2011a). On average, each PT owner cultivates 11–15 hectares of land, and given the average farm size in Bangladesh is only 0.59 ha, this implies that all of the PT owners are capable of providing tilling services to other farmers after completing their own tasks. Since the 1990s the use of threshers and other machines has also increased significantly. For example, at present a total 0.26 million threshers and 1.25 million sprayers are engaged in serving 14.87 million farm households in Bangladesh (Bangladesh Bureau of Statistics [BBS], 2010).

Bangladesh continues to import significant numbers of agricultural machinery (*Mottaleb et al.*, 2016), mainly from China (Table 1). The table shows that in the last decade on average a total of 106 importers imported 50,551 PTs annually at an average price of Bangladesh Taka (BDT) 68,163 (equivalent to USD959) per unit.^2^ To increase PT adoption, the GOB still provides a nearly 25 per cent subsidy to a limited number of targeted farm households in targeted communities.

**Table 1.** Import of power tillers (PTs), Bangladesh, 2004–2013

			Unit price of power tiller (PT)^a^	
Year	No. of importers	No units imported	In BDT	In USD^b^
2004–05	109	54,675	49,390	804
2005–06	99	52,863	56,346	839
2006–07	77	37,606	56,618	820
2007–08	106	56,460	59,100	861
2008–09	103	55,604	73,334	1,065
2009–10	119	44,872	69,086	998
2010–11	113	70,843	76,570	1,075
2011–12	116	51,266	85,356	1,079
2012–13	112	30,771	87,671	1,097

*Source*: GOB (2014). ^a^ Price is calculated as the import value plus 2.01 per cent import tax. ^b^ Converted using yearly average exchange between BDT and USD.

In Bangladesh, diesel engines and PTs are used for numerous economic activities. For example, the use of low horsepower diesel engines is common for manoeuvring fishing trawlers and boats and PTs are used for road transport by attaching a flatbed trailer. People also use small diesel engines for milling rice and grinding wheat, setting the engine on locally-made mobile milling machines with entrepreneurs moving from house to house. This extra machine usage enables the lead farmers/service providers to earn extra income.

## 3. Data and analytical methods

### 3.1. Sampling and data used

**Figure 2.**
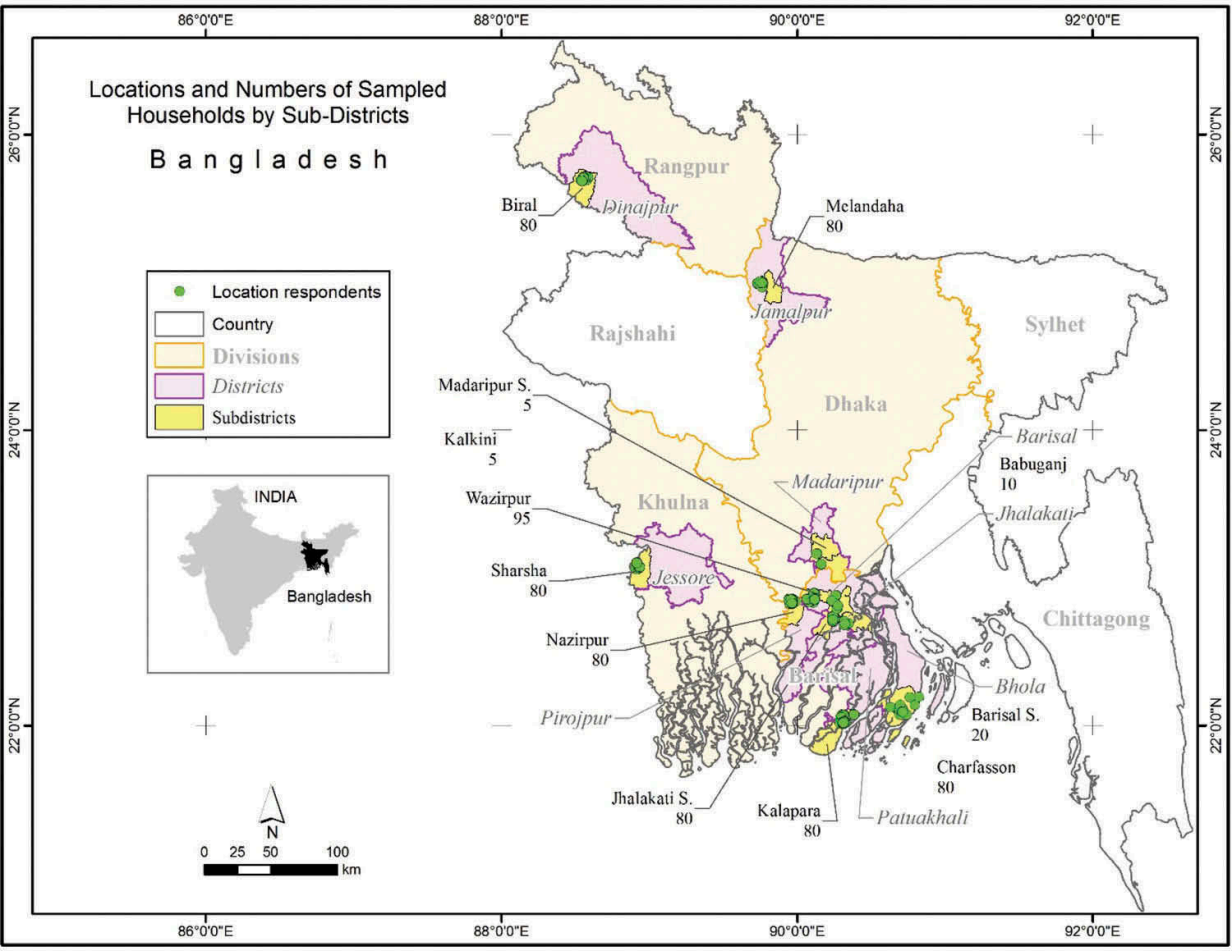
Survey locations and the numbers of sampled households by sub-dsitricts. *Source*: Survey, 2015.

This study is based on primary data collected in 2015 from 695 randomly-selected farm households located in four divisions, nine districts, 12 sub-districts, 15 unions and 43 villages (Figure 2). The survey focuses on Barisal Division, one of the most impoverished Divisions in Bangladesh (BBS, 2011b), and with a relatively low cropping intensity (defined as the number of crops grown per calendar year in a specific land area) compared to the national average (*MoA and FAO*, 2013). In addition to Barisal Division, the survey area includes (sub-) districts of Dhaka, Khulna, and Rangpur divisions (Table 2).

**Table 2.** Sampled respondent by location

Name of the division	Name of the district	Name of the sub-district	No. of total sampled households	No. of PT tilling service providers among sampled households
Barisal	Barisal	Babuganj	10	3
		Barisal sadar	20	3
		Wazipur	95	10
	Bhola	Charfassion	80	17
	Jhalokathi	Jhalokathi sadar	80	5
	Patuakhali	Kolapara	80	6
	Pirojpur	Najirpur	80	4
Dhaka	Jamalpur	Melandaha	80	8
	Madaripur	Madaripur sadar	5	1
		Kalkini	5	1
Khulna	Jessore	Sharsha	80	11
Rangpur	Dinajpur	Birol	80	2
Total	9	12	695	71

*Source*: Survey, 2015

The survey focused on collecting information from service providers and their client farmers. In the sample design we focused on irrigation services in the dry *Rabi* (winter) season. Using information from the BADC (2013), the sampled (sub-)districts were selected based on the criterion that the selected (sub-)districts were equipped with the highest numbers of irrigation service providers. Using the same criterion, the unions and villages are selected in the sampled sub-districts. To randomly select the pump owners after selecting the survey village, this study used information provided by the lowest level of public agricultural extension officers (Sub-Assistant Agriculture Officer, SAAO) on the number of pump owners, as such information is not available at the union and village level from any secondary source. From each sampled sub-district, one union and one to four villages were selected. Based on the lists of the irrigation service providers provided by SAAOs, four irrigation service providers in a sampled village, and four client farmers for each irrigation service provider were randomly selected as the respondents. Thus, in general, from every sampled village, 5–20 farm households (1–4 irrigation service provider and 4–16 of their client farmers) were interviewed for information collection.

Table 2 presents detailed sample information. Out of the total of 695 farm households, 71 owned a PT and all offered PT tilling services to client farmers – making households with PT ownership and those providing PT tilling services identical in our study. In the next subsection, analytical models are developed to first characterise farm households that invested in PTs and provide PT tilling services to client farmers; and second, to assess the factors that affect the fee-for-service charges.

### 3.2. Model specification

To examine the factors that influence the decision of a household to purchase a PT and provide tilling services to other farmers in Bangladesh, Equation (1) is developed as follows:

(1) $$Y_{1i}=\alpha_{0}+(HHC_{i})\emptyset +\alpha_{1}\left(Remittance_{i}\right)+\alpha_{2}\left(Credit_{i}\right)+\alpha_{3}\left(Borrowed_{i}\right)+\alpha_{4}\left(Risk_{i}\right)+\sum_{d=1}^{11}\beta_{j}(SD_{j})+\varepsilon_{i}$$

where *Y_1i_* is a dependent variable that assumes the value 1 if a household owned a PT and provided services to others, and 0 otherwise. Among the explanatory variables, *HHC_i_* is a vector of independent variables that includes age and years of schooling of the household head, the major occupation dummy that assumes a value of 1 if a household head is engaged in non-farm activities as a major source of livelihood and 0 otherwise, and the total number of male family members in the household. $Remittance_{i}$ is a dummy variable (1 if yes, 0 if no) for households which receive remittances from extended family members living in cities and abroad, *Credit_i_* a self-perceived credit constraint dummy which assumes the value 1 if a household head perceived himself as facing credit constraint and 0 otherwise; $Borrowed_{i}$ is the amount of money that PT owner either actually borrowed or could borrow from formal sources, and $Risk_{i}$ is the self-assessed score regarding general risks whose value ranges between 0 at the minimum if a household head is entirely risk averse, and 10 at the maximum if a household head is entirely not averse to risk taking. The independent variable also includes 11 dummies for 12 sampled sub-districts $\left(SD_{j}\right)$to capture the sub-district level of unobserved influences affecting ownership and providing tillage service by owning a PT by the sampled households. In this case, Birol sub-district of Dinajpur District of Rangpur Division was set as a base (assigned the value 0). It is expected that a risk-taking household head, with more years of schooling and endowed with more male family members, is more likely to be a tilling-service provider compared to others.

In the case of Equation (1), the dependent variable is a binary response variable (0, 1), and thus to estimate the probability of PT ownership and providing tillage services by a household, a maximum likelihood estimation procedure applying a probit model estimation method is applied. In estimating the probability of PT ownership and providing tilling services, five models were estimated. Out of 695 sampled households only 71 owned PTs, all of whom provide tilling services to other farmers. The first model includes the full sample. However, the ubiquity of the households with no PT (*Y_i_ = 0*) might divert the estimated results toward biasness. To enhance the balance between yes (*Y_i_ = 1*) and no (*Y_i_ = 0*) and to check the robustness of the major findings, four additional models are specified. First, four groups of data sets are randomly generated with an almost equal number of observations, each of which includes 167–173 non-PT owner observations. Second, the 71 PT owner observations (*Yi *= 1) are added to each set, and the models are estimated applying a probit model estimation procedure. By doing this, this study checked the sensitivity of the major findings as well as the robustness of the major policy variables.

Under the fee-for-tilling-service system, service providers charge fees to the client farmers for providing PT tilling services. To examine the factors that affect the service charges, Equation (2) is developed as follows:

(2) Y2i=θ0+(HHCi)Φ+θ1(Yearsi)+θ2(Sifengi)+θ3(Enginehorsepoweri)+θ3(Manageri)                                                         +∑k=111ϕk(SDk)+θ4(λi^)+ξi

where *Y_2i_* is a dependent variable that includes the service charge per hectare for one full tillage in BDT. Among the explanatory variables in the Equation (2), *HHC_i_* is a vector of independent variables that includes all the household level variables described in the case of Equation (1). However, *HHC_i_* also includes an experience variable that is the years a household head has been engaged in the tilling-service business. $Sifeng_{i}$ is a brand dummy that assumes the value 1 if the PT model is Sifeng, or 0 otherwise; $Manager_{i}$is a hired manager dummy that assumes the value 1 if a household hires a manager/worker to operate the PT, or 0 otherwise. Similar to Equation (1), the independent variable includes 11 dummies for 12 sampled sub-districts in which Birol sub-district of Dinajpur District of Rangpur Division is set as the base. Equation (2) also includes λi^, a generalised inverse Mills ratio calculated from Equation (1) following the estimation process suggested by Vella (1998), as an independent variable. Both in Equation (1) and (2), *α*_0_ $and\theta_{0}$ are the scalar parameters, and *ϕ* ,Φ, *α*,θ,βandϕare the parameters to be estimated; *I* stands for household, and εarethe random error terms. Note that as Equation (2) includes λi^ estimated from Equation (1), to correct for the standard error, we applied the bootstrapping method and replicate the regression estimation procedures 1000 times in the case of Equation (2). Finally, three different model versions are estimated, depending on the inclusion of two additional variables: the engine horsepower (HP) of the PT engine and an interaction term between the PT, HP and hired manager.

To estimate Equation (2), the tobit estimation approach is applied censoring at 0 (in the left), as out of 695 sampled farm households, only 71 of the respondents provide tillage services. As only 71 PT owners provide PT services on a fee basis, applying OLS estimation in this case might generate biased results.

## 4. Results and discussion

### 4.1. General findings

Two major models of PT dominate the Bangladesh market: Sifeng and Dongfeng brands. The Dongfeng PT is produced by Shanghai Changzhou Dongfeng Agricultural Machinery Group Co., China, and the Sifeng PT is produced by Zhejiang Sifeng Group Zhejiang Province, China. Based on the information from one of the major importers, the retail price of the Dongfeng brand 12, 16 and 20 horsepower (HP) PTs are USD1400–1435, USD1487–1490 and USD1560 respectively. On the other hand, the retail price of the 12, 16 and 20 HP Sifeng PTs are USD1487–1538, USD1500–1564, and USD1615, respectively. During the survey the enumerators requested the PT service providers for their opinion on why the Sifeng PT is relatively more costly than the Dongfeng PT. The majority of the sampled respondents replied that irrespective of the engine horsepower, the Sifeng PTs are relatively heavier than the Dongfeng PTs which provides an extra advantage in puddling in wetlands particularly for preparing irrigated *Boro* rice fields.

Out of 695 sampled farm households, a total of 71 (10%) owns a PT and provide tilling services to other farmers. In general, because of the high initial cost, and the availability of service providers, only a minority of farm households has purchased a PT. Table 3 presents the basic characteristics of the sampled households based on whether or not a farm household owns a PT and provides tillage services to farmers in the *Rabi* season 2014–2015. As expected, households that provide PT tilling services are also likely to be more endowed compared to others – not only do they have a PT, they are also more likely to be equipped with other agricultural machinery, such as irrigation pumps and rice and wheat threshing machines (Table 3). A PT tilling service provider is also likely to have more land and livestock, more schooling, a larger family, and more likely to have received remittances from their extended household members who are living in cities or abroad compared to non-service providers.

**Table 3.** Basic information of the sampled households

Variable	Not a tilling service provider	Tilling service provider	Mean difference, t-statistic and the level of significance
	Mean (a) (Std. Dev)	Mean (b) (Std. Dev)	
Age, household head	45.31	44.14	1.17
	(12.77)	(11.97)	(0.73)
Years of schooling, household head	4.88	6.23	−1.34***
	(4.72)	(4.71)	(−2.28)
% Operator with non-farm job as major income source	7.70	2.8	4.9
	(27.0)	(16.7)	(1.50)
No. of family members	4.76	5.17	−0.41**
	(1.60)	(1.96)	(−1.99)
No. of male family member	2.44	2.80	−0.36***
	(1.09)	(1.42)	(−2.54)
No. of adult male members	1.60	1.76	−0.16*
	(0.85)	(0.93)	(−1.45)
No. of adult female members	1.54	1.49	0.05
	(0.73)	(0.79)	(0.50)
Land cultivated (ha)	0.83	2.11	−1.28***
	(0.73)	(2.60)	(−9.47)
% Household received remittance	12.5	21.1	−0.09**
	(33.0)	(41.1)	(−2.-3)
% Households under credit constraint	54.3	42.3	12.1**
	(50.0)	(49.7)	(1.93)
Amount actually borrowed or could borrow from formal credit organisations (‘000, BDT)	30,760	39,037	−0.81
	(3)	(7844)	(0.21)
Self-rated risk score, operator	6.27	7.17	−0.90***
	(2.24)	(2.26)	(−3.19)
% Household equipped with an irrigation pump	38.8	78.9	−40.0***
	(1.9)	(4.9)	(−6.66)
% Household equipped with a thresher machine	7.21	40.8	−33.6***
	(1.03)	(5.90)	(−9.21)
No. of cows and buffaloes owned	0.003	1.08	−1.08***
	(0.002)	(0.03)	(−83.06)

*Source*: Survey, 2015

*Notes*: Differences = Mean (a) – Mean (b). H_0_: Diff = 0, H_1_: Diff<0 (one sided t-test). ***, ** and * indicate the 1 per cent, 5 per cent, and 10 per cent levels of significance, respectively.

During the survey the sampled household heads were requested to rate themselves as a risk-averting or risk-taking person by numbering themselves between 0 (in the case of someone who completely avoids any type of risk) and 10 (in the case of someone who almost seeks out risks). The sampled respondents were also asked to indicate whether or not they are under any type of credit constraint. Interestingly, households that own a PT and provide tilling services are more likely to describe themselves as risk-taking and less likely to be credit constrained (Table 3). On average, a household borrowed or could borrow BDT 30–40,000 from the formal monetary organisations, with no significant difference between households based on PT ownership.

**Figure 3.**
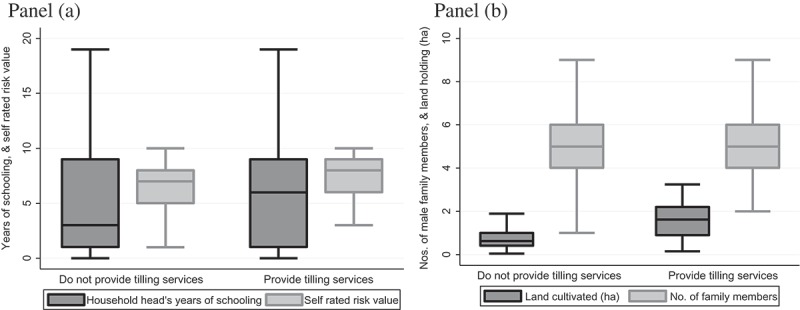
Selected indicator contrasts for households providing PT tilling service versus those that do not. Panel (a) 1. household head’s years of schooling; 2. self-rated value related to risk aversion/preference; (b) 3 land cultivated (ha); and 4. Number of family members (box plots excluding outliers). *Source*: Survey, 2015

In summary, households that provide PT tilling services are more endowed with both human and physical capital compared to other households (Figure 3). Still, this does not imply a causal relationship among variables of interest and tilling-service provision by the sampled households, an issue elaborated below in relation to the econometric model.

Table 4 provides some of the specific features exclusively related to the PT tilling-service-providing households. Nearly 55 per cent of the service providers has been providing such services for more than seven years on average. Two-thirds of the service providers operate 12 HP PTs and overall the market share of Sifeng PTs is 56 per cent.

**Table 4.** Specific information on the tilling service provider

Variable	Frequency (%)	F-statistic (Prob > F)
*Years in tilling service business*		
Up to 3 years	21 (29.6)	2.81***
4–6	10 (14.1)	(0.00)
7–10	15 (21.1)	
>10	27 (35.2)	
*Machine horsepower (HP)*		
12	48 (67.6)	2.61***
16	14 (19.7)	(0.00)
20	9 (12.7)	
*Model*		
Sifeng	40 (56.3)	2.47**
Dongfeng	28 (39.4)	(0.01)
Other	3 (4.2)	
Hired a manager	45 (63.4)	1.91**
		(0.05)
*Payment method for hired manager*		
Daily basis	31 (77.8)	1.34
Share of earnings	8 (17.8)	(0.15)
Seasonal contract	6 (13.3)	
*Total land served (ha cultivated) in 2014–15 dry Rabi season*		
Up to 5	14 (19.7)	16.19*
6–15	24 (33.8)	(0.08)
>15	33 (46.5)	
*Service charge (one full tillage, BDT ha^−1^)*		
Up to 2,000	31 (43.4)	11.95***
2,001–2,500	16 (22.5)	(0.00)
>2,500	24 (33.8)	
*Total revenue PT tilling service 2014/15 Rabi season (BDT)*		
Up to 50,000	28 (39.4)	6.49***
50,001–100,000	14 (19.7)	(0.00)
Above 100,000	29 (40.9)	

*Source*: Survey, 2015.

*Notes*: H_0_: Mean(group _1_) = – – = Mean(group _n_),***, ** and * indicate the 1 per cent, 5 per cent, and 10 per cent levels of significance, respectively. Grouping of the respondents is done based on the unions they are located.

PT operation is customarily performed by men in Bangladesh. Interestingly, 63 per cent of the service providers hired a paid worker to operate their PT during the peak season. PT owners tend to pay hired PT managers on a daily basis (78%), with daily payments of BDT 350–450 (USD4.49–5.77). A few PT owners offer earning shares to the appointed hired managers (25–30% of revenues), and a few offered seasonal contracts (BDT5,000–13,000 [USD64–167]).

Nearly half the PT owners serviced 15 hectares or more land in the 2014–2015 Rabi season. Two-thirds charged up to BDT 2500 per ha (USD32) for a full one-time tilling service and three-fifths earned up to BDT100,000 (USD1280) by providing PT tilling services in the 2014–2015 dry *Rabi* season. Service charges are similar for establishing dry land crops, such as wheat, maize and vegetables, although thorough land levelling may imply extra charges.

### 4.2. PT ownership and tilling service provision

Table 5 presents the estimated functions explaining PT ownership and tilling service provision by a farm household (models 1–5). The first model uses the full set of observations (695), while models 2–5 use a random subset of observations. Among the demographic variables, years of schooling of the household head positively influenced PT acquisition and tilling services. Schooling might provide more off-farm income earning opportunities and thereby enhance the capacity to invest in costly agricultural machinery; but the non-farm occupation dummy shows that household heads working in the non-farm sector are actually more reluctant to invest in agricultural machinery. Education seems important simply because it facilitates access to market information and foreseeing the PT tilling service as a profitable business opportunity. Moreover, education may enhance agricultural extension linkages, and facilitates access to information and acquiring PTs at subsidised rates. In fact, among 71 sampled PT service providers, at least two of them received PTs at a subsidised rate, and both household heads were relatively more educated than the others. To develop agricultural machinery services elsewhere, policy-makers and donor agencies should primarily target potential investors/service providers from within the farmer community and particularly should target relatively educated household heads who are endowed with the necessary assets. It is unlikely that economically affluent non-farm households will invest in agricultural machinery to provide tillage services. Importantly, the number of male family members also positively influences PT ownership and tilling services across the estimated functions. In Bangladesh, PT operation is customarily performed by men and availability of male family members may facilitate PT acquisition and setting up a tilling-service business.

**Table 5.** Estimated functions applying probit estimation approach explaining tilling service provision in Bangladesh

Dependent variable	Provide PT tilling services (yes = 1)				
Observation group	Full data set	Randomly generated observation group			
Model specification	Model 1	Model 2	Model 3	Model 4	Model 5
Age, household head	−0.01	−0.01	−0.0003	−0.01	−0.005
	(0.01)	(0.01)	(0.01)	(0.01)	(0.01)
Years of schooling, household head	0.03**	0.05**	0.011	0.06***	0.04**
	(0.01)	(0.02)	(0.02)	(0.02)	(0.02)
Non-farm sector major occupation household head (dummy, yes = 1)	−0.79**	−1.32***	−1.06**	−1.31***	−1.27***
	(0.32)	(0.41)	(0.43)	(0.33)	(0.46)
Total male family member	0.13**	0.19**	0.26***	0.14*	0.11
	(0.06)	(0.08)	(0.08)	(0.08)	(0.07)
Remittance receiving household (dummy, yes = 1)	0.31*	0.76***	0.36	0.42	0.44*
	(0.18)	(0.25)	(0.23)	(0.27)	(0.24)
Credit constrained household (dummy, yes = 1)	−0.24*	0.62***	0.50**	0.46**	0.32*
	(0.14)	(0.20)	(0.19)	(0.19)	(0.19)
Total amount borrowed from formal organisations (000, BDT)	0.001	0.001	0.00055	0.0015	−0.001
	(0.00)	(0.00)	(0.00)	(0.00)	(0.00)
Self-rating about risk aversion (0 completely avert risk, 10 completely adopt risk)	0.095**	0.08*	0.070*	0.067	0.098**
	(0.04)	(0.05)	(0.04)	(0.05)	(0.04)
					
Babuganj subdistrict (dummy)^a^	1.48***	1.61***	1.43**	1.60**	1.33**
	(0.50)	(0.60)	(0.60)	(0.67)	(0.59)
Barisal sadar subdistrict (dummy)	0.94**	0.91	1.23*	1.09**	1.18**
	(0.47)	(0.56)	(0.63)	(0.55)	(0.57)
Char Fassion subdistrict (dummy)	1.25***	1.25***	1.45***	1.75***	1.47***
	(0.34)	(0.43)	(0.44)	(0.43)	(0.42)
Jhalokati sadar subdistrict (dummy)	0.36	0.29	0.77	0.32	0.64
	(0.39)	(0.46)	(0.50)	(0.47)	(0.49)
Kalkini subdistrict (dummy)	0.99	0.21	0.91	0.73	1.09
	(0.63)	(0.68)	(0.63)	(0.86)	(0.86)
Kolapara subdistrict (dummy)	0.51	0.72	0.96**	0.21	0.57
	(0.36)	(0.46)	(0.47)	(0.45)	(0.44)
Madaripur sadar subdistrict (dummy)	1.28*	0.82		1.08	0
	(0.69)	(0.87)		(0.92)	(.)
Melandaha subdistrict (dummy)	0.79**	0.97**	1.43***	0.52	0.95**
	(0.36)	(0.45)	(0.45)	(0.45)	(0.43)
Najirpur subdistrict (dummy)	0.30	0.57	0.74	−0.011	0.51
	(0.39)	(0.50)	(0.49)	(0.52)	(0.50)
Sarsha subdistrict (dummy)	0.96***	0.98**	1.26***	1.28***	1.34***
	(0.34)	(0.44)	(0.44)	(0.45)	(0.45)
Wazirpur subdistrict (dummy)	0.73**	0.96**	1.45***	0.82*	1.21***
Constant	−3.06***	−2.49***	−3.00***	−1.93***	−2.53***
	(0.48)	(0.65)	(0.63)	(0.57)	(0.55)
Observations	695	244	244	245	244
Wald Chi (19)	57.04	59.75	41.49	77.66	44.74
Probability> Chi^2^	0.00	0.00	0.00	0.00	0.00
Pseudo R^2^	0.13	0.19	0.14	0.22	0.15
Log pseudolikelihood	−199.90	−127.66	−136.90	−126.06	−131.50

*Notes*: Numbers in parentheses are robust standard errors. * is significant at the 10 per cent level, ** is significant at the 5 per cent level and *** is significant at the 1 per cent level. ^a^ Subdistrict base = Birol, Dinajpur district, Rangpur division.

The probability of PT ownership and tilling services is higher in the case of households that receive remittances, although only significant in model 1 and 2 of the four models with the data subsets (Table 5). In contrast, the probability of PT ownership and tilling services declines if a farm household faces credit constraints (model 1, 2 and 5 – Table 5). A PT is costly and requires a substantial investment (Table 1), so credit access can lessen capital constraints; whereas credit access constraints reduce the likelihood of PT acquisition and tilling services. One might argue that in addition to credit access (dummy), the actual amount of money that a farm household borrowed might affect the PT acquisition and tilling services, but these had no significant influence. In contrast, the risk-taking attitude of the household head positively influences PT ownership and tilling services, a robust finding across all estimated models (models 1–5, Table 5). This implies that policies aimed at the development of service providers should target households who are ready to take the investment risks associated with new agricultural technologies.

The sub-district dummies clearly show that households in sub-districts in Barisal Division (Babuganj, Barisal Sadar, Char Fassion, and Wazirpur) are equipped with more PT owners and service providers than Birol sub-district (of Dinajpur District, base). However, PT ownership and service provision in Jhalokathi Sadar, Kalkini and Nazirpur sub-districts (the coastal sub-district) is low compared to Birol. Special government and donor support can enhance PT ownership in the coastal region to encourage farmers to invest in agricultural machinery despite the vulnerability to weather-related risks.

### 4.3. PT service charges

Table 6 presents estimated functions to explain PT tilling service charges. The main model 1 shows that household head education inflates the service charge, as do the number of male family members and whether a manager is hired. Education may facilitate service price setting and bargaining power. As mentioned above, PT operation and land preparation are customarily mainly done by males linked to the perceived nature of physical work and social mobility. Consequently, households with more male family members are not only more likely to own PTs than others (Table 5), but also more likely to receive higher tilling service charges than others (Table 6), perhaps linked to having more bargaining power and their overall tilling service business model. A hired manager to operate the PT entails costs for the PT owner under the prevailing daily wage rate model, and it seems logical to recover a part of the costs by charging more for their services than others.

**Table 6.** Estimated functions applying censored (left) tobit estimation approach explaining service charges (‘000, BDT ha^−1^) in Bangladesh

Dependent variable	Service charge received (‘000, BDT ha^−1^)		
Model specification	Model 1	Model 2	Model 3
Age, household head	−0.011	−0.016	−0.010
	(0.01)	(0.01)	(0.01)
Years of schooling, household head	0.078**	0.054*	0.046
	(0.03)	(0.03)	(0.04)
Non-farm sector as major occupation household head (dummy, yes = 1)	−1.34	−0.86	−0.82
	(1.17)	(1.14)	(1.02)
Total male family member	0.20**	0.10	0.061
	(0.09)	(0.11)	(0.12)
Hired manager (dummy, yes = 1)	0.49*	0.23	2.92*
	(0.27)	(0.30)	(1.60)
Years in soil tilling service business	0.016	0.020	0.020
	(0.02)	(0.02)	(0.02)
Sifeng model (dummy, yes = 1)	0.20	0.10	−0.032
	(0.30)	(0.31)	(0.27)
Generalised inverse Mills ratio (GIMR)	2.50***	1.81***	1.22
	(0.27)	(0.36)	(0.75)
Engine horse power		0.12**	0.22
		(0.05)	(0.14)
(Engine horse power) X (Hired manager)			−0.21
			(0.14)
Babuganj subdistrict (dummy)	2.03	1.68	1.34
	(1.55)	(1.35)	(1.22)
Barisal sadar subdistrict (dummy)	1.67	1.66	1.73
	(1.51)	(1.33)	(1.19)
Char Fassion subdistrict (dummy)	0.69	0.21	0.36
	(1.41)	(1.26)	(1.14)
Jhalokathi Sadar subdistrict (dummy)	0.59	0.99	1.18
	(1.40)	(1.20)	(1.17)
Kalkini subdistrict (dummy)	1.63	1.68	1.39
	(2.19)	(1.91)	(1.58)
Kolapara subdistrict (dummy)	−0.53	−0.42	−0.15
	(1.43)	(1.22)	(1.14)
Madaripur sadar subdistrict (dummy)	3.43	3.35	3.31
	(2.61)	(2.42)	(2.20)
Melandaha subdistrict (dummy)	−0.33	−0.30	−0.34
	(1.40)	(1.20)	(1.09)
Najirpur subdistrict (dummy)	−0.36	−0.0034	0.22
	(1.52)	(1.30)	(1.26)
Sarsha subdistrict (dummy)	0.19	0.12	0.023
	(1.46)	(1.29)	(1.20)
Wazirpur subdistrict (dummy)	0.98	1.17	1.36
	(1.38)	(1.19)	(1.12)
Constant	−3.09*	−2.75*	−3.13**
	(1.70)	(1.49)	(1.54)
Sigma	0.83***	0.80***	0.76***
	(0.14)	(0.14)	(0.15)
Observations	695	695	695
Wald Chi (19)	298.23	308.14	159.10
Probability> Chi^2^	0.00	0.00	0.00
Pseudo R^2^	0.73	0.75	0.76
No. of left censored observations	624	624	624
Log likelihood ratio test (assumption model 1 is based in model 2) LR chi^2^ (1)	(8.32)		19.46
P>Chi^2^	0.04		0.00

*Notes*: Numbers in parentheses are bootstrapped standard errors replicated for 1000 times. * is significant at the 10 per cent level, ** is significant at the 5 per cent level and *** is significant at the 1 per cent level. ^a^ Subdistrict base = Birol, Dinajpur district, Rangpur division.

A number of other variables had no influence on the service charges, including the years in the PT tilling service business, the particular PT model, the age of the household head or a primary non-farm income source. Perhaps somewhat surprising too, none of the district dummies had a significant effect.

To capture any kind of biasness in the PT ownership by households we also included the Generalised Inverse Mills Ratios (GIMR). The GIMR indicates a positive correlation between the unobservable characteristics in PT ownership and the service charge received, thus indicating that unobserved characteristics of the service providers increase the tilling service charges.

In model variants 2 and 3 (Table 6), this study sequentially added two more variables: engine horsepower (model 2 and 3) and an interaction term constructed by multiplying engine HP with the hired manager dummy (model 3). Model 2 shows that service providers with higher HP PT charge more than others on average, likely associated with the higher PT investment cost and enhanced tillage operations (for example faster tillage). Interestingly, the hired manager dummy ceases to be significant in model 2 suggesting an interaction between the engine HP and hired manager. Model 3 therefore includes such an interaction term and the model than reverts to an even more pronounced hired manager effect, whereas neither the engine HP nor the interaction effect is significant.

## 5. Conclusion and policy implications

While farm mechanisation is imperative in developing countries for sustainable agricultural development to ensure food security and enhance livelihoods, there is no agreement on how best to mechanise smallholder farms and ensure wider access to agricultural machinery by resource-poor farm households. Using the highly-successful Bangladesh mechanisation experience as a case, the present paper has demonstrated that supportive government policies along with private initiatives are instrumental in facilitating farm mechanisation and ensuring wider access to agricultural machinery even by marginal farmers.

Analysing historical events and government policy, the present paper demonstrates that the market liberalisation policy of the GOB by removing tariff and non-tariff barriers in the early 1990s was the initial step to facilitate agricultural mechanisation in Bangladesh. By allowing imports of scale-appropriate, relatively-cheap agricultural machinery, mainly from China, the government provided the initial boost and stepping stone to farm mechanisation in Bangladesh. In view of new opportunities, lead farmers started investing in irrigation pumps and PTs first primarily for their own crops. Later on, realising the provision of agricultural machinery services as a profitable business, these lead farmers started to provide machinery services to other farmers on a fee-for-service basis to earn extra income. Because of the pervasiveness of the service-provision system in Bangladesh agriculture, at present 55 per cent of the total farmland in Bangladesh is under irrigation, and 80 per cent of the total cropland is cultivated using PTs while the associated machinery ownership is much less widespread.

There is scant evidence on who the service providers are (for example, Sims, *Rottger, & Mkomwa*, 2011). Using primary data collected from 695 farm households in Bangladesh, the present paper, thereby characterised the agricultural-machinery-service providers using the PT tilling service as a case. The present paper clearly demonstrates that the risk-taking household heads with relatively more years of schooling and who are primarily engaged in agriculture as their major occupation, are more likely to invest in agricultural machinery and provide machinery services to other small farmers on a payment basis. The findings indicate that in developing countries in general education must be ensured; however, for the farmers who cannot go back to school, an awareness programme and training can be arranged to supplement formal education. Education may encourage farm households to take calculated risks related to agricultural machinery investments. Empirical results also confirm that credit availability positively affects the decision to purchase a PT and provide services; as does the receipt of remittances, male family members, having farming as the main income and locational factors. On the other hand, general education, a hired manager, male family members and unobserved family characteristics shape the service charge received by the service-providing household.

The role of credit access reiterates the need to facilitate credit access for resource-constrained farm households to reduce capital constraints to encourage them to invest in costly agricultural machinery. International donor agencies, along with local government and private development partners, can play an important role in ensuring wider credit access at accessible interest rates for farm households in developing countries. In addition, although this study does not focus on this issue directly, investment in basic infrastructure and market access can reduce investment risk, and thus might enable farm households to invest in machinery (for example, *Mottaleb et al.*, 2016).

Finally, it is important to note that although an estimated one third of the Bangladesh agricultural labour force is female (for example, *Rahman*, 2000), women in Bangladesh are less likely to own or operate productive agricultural machinery (for example, *Mottaleb & Krupnik*, 2015). Social inclusiveness of women into mainstream agricultural machinery ownership and machinery service provision remains a real challenge, and future research should address this particular issue.

## Notes

1. Total gross annual crop area (that is the aggregate area across seasons, including land sown more than once) was 14.41 million hectares, net cropped area (that is actual crop land) was 7.94 million hectares in 2010 (BBS, 2011a).

2. Currently USD 1=78 BDT, approximately.
